# Phytochemical, Antioxidant and Mitochondrial Permeability Transition Studies on Fruit-Skin Ethanol Extract of *Annona muricata*

**DOI:** 10.1155/2019/7607031

**Published:** 2019-12-31

**Authors:** Wisdom O. Iyanda-Joel, Olusayo B. Ajetunmobi, Shalom N. Chinedu, Emeka E. J. Iweala, Oluwatobi S. Adegbite

**Affiliations:** ^1^Department of Biochemistry, College of Science and Technology, Covenant University, Ota, Nigeria; ^2^Institute of Integrative Biology, University of Liverpool, Liverpool, UK

## Abstract

Uncontrolled cell proliferation hallmarks cancer and most cancer cells have developed multiple resistance to the drugs employed for their treatment. The study examined the phytochemical and antioxidant properties of the fruit-skin ethanol extract of *Annona muricata* Linn. (ESA) and its effect on rat liver mitochondrial membrane permeability transition (MMPT). Qualitative phytochemical study and antioxidant assays were carried out following established protocols while the opening of the MMPT pore in the presence of varying concentrations of the extract was assayed spectrophotometrically under succinate-energized conditions. Calcium chloride (CaCl_2_) and spermine were used to trigger and inhibit pore opening respectively. Cytochrome c release was assayed for using ELISA kit. Terpenoids, steroids, phenols among other phytochemicals were found present in ESA and the extract showed very low antioxidant properties at the tested concentrations based on the diphenyl-1-picryhydrazyl (DPPH) radical scavenging activity assay. Lipid peroxidation was induced in a concentration-dependent manner on both the cytosolic and mitochondrial hepatocyte fractions *in vitro*. In the absence of CaCl_2_ 0.84 mg/mL concentration of ESA induced MMPT pore opening by 129% while the extracts showed no inhibitory activity in its presence. The induction fold corresponded with the concentrations of cytochrome c released. The fruit-skin ethanol extract of *Annona muricata* at certain concentrations may possibly contain bioactive compounds that induce apoptosis.

## 1. Introduction

**Figure 1:**
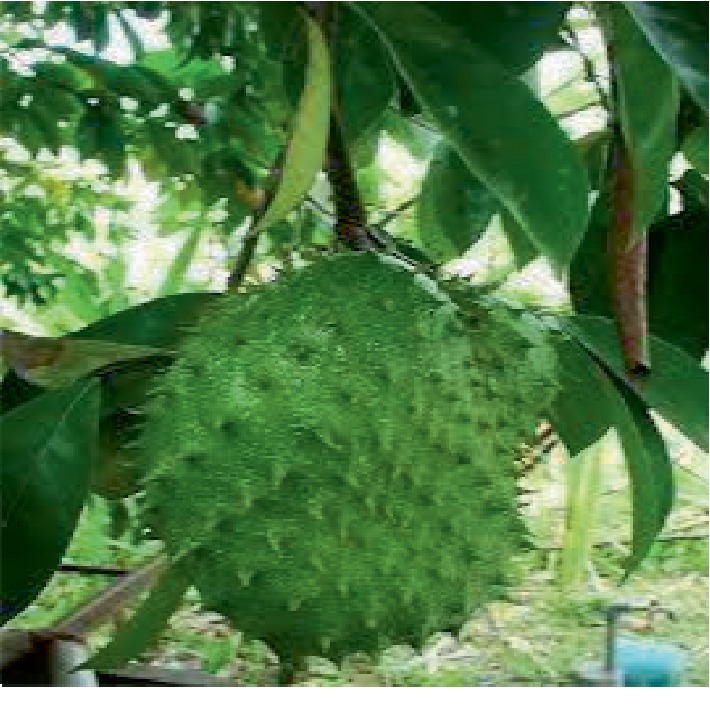
*Annona muricata* fruit on the tree.

Embedded in nature is all that is needed to tackle the myriad infectious and noninfectious diseases of ancient prevalence and those of current emergence. With well above 80% of the global population counting on trado-medicines (TMs) for their healthcare, the need therefore arises to thoroughly investigate the flora in order to develop nature-friendly therapeutics thereby finding a lasting solution to these global agelong burdens of terminal diseases [1–4]. Two of such agelong burdens are infectious diseases and cancers which are the leading causes of death globally and constitute predominant threat to public health [5]. There is a significant connection between these two classes of diseases due to the fact that etiological agents of infections are accountable for approximately 25% of all cancer incidence; and pathogen invasion as well as malignant tumors develop as a result of compromised innate immune defense mechanisms [6, 7]. Furthermore, with the increasing threat trends that antimicrobial drug resistance poses globally, most novel therapies for cancer now target all cells undergoing division and these are accompanied by deleterious side effects [5, 8, 9]. Moreover, the cytotoxic activity of many of these drugs are mediated via the intrinsic cell death pathway, where mitochondria play a very crucial role. *Annona muricata* Linn. (Figure 1) also known as soursop is one of the species from the family *Annonaceae* and has been remarkably reported to possess potent selective anticancer activities through the specific action of a group of phytochemicals called Annonaceous acetogenins, ACGs [10]. More recently, Moghadamtousi and research group team (2014) uncovered the action mechanism of the *A. muricata* leaf extract in suppressing the migration and invasion of cancer cells via mitochondrial membrane disruption thereby arresting G_0_/G_1_ phase cells and inducing apoptosis [10, 11]. However, some researchers reasoned that the selective cytotoxic activity of *A. muricata* extracts and purified compounds may be due to excessive demands of ATP in cancerous cells [12]. Nonetheless, this plant still remains as one of the crops for the future; although it has been thoroughly explored in the last decade against several cancer cell lines and pathogenic bacteria [13]. This current study was poised at examining the phytochemical and antioxidant constituents of the fruit-skin ethanol extract of *A. muricata* vis à vis its opening of the MMPT pore which is the hallmark of mitochondrial apoptosis and crucial to annihilation of pathogenic and cancer cells.

## 2. Materials and Methods

### 2.1. Materials

Diphenyl-1-picryhydrazy (DPPH), trichloroacetic acid (TCA), thiobarbituric acid (TBA), mannitol, sucrose, N-(2-hydroxyethyl) pipearizine-N-(2-ethanesulfonic acid) (HEPES), rotenone, spermine, bovine serum albumin (BSA),standard solution, standard diluent, chromagen A and B, anti-cytochrome C antibodies labelled with biotin, streptavidin-HRP, stop solution, 30X wash solution and all other reagents were purchased from Sigma Chemical Co. (St. Louis, MO, USA) and were of the highest purity grade. Healthy male Wistar strain albino rats (weighing between 120 and 160 g), purchased from the Federal University of Agriculture, Abeokuta (FUNAAB), Nigeria were fed with standard commercial diets and water *ad libitum* and handled in accordance with the WHO Good Laboratory Practice (GLP) regulations throughout the experiment period.

### 2.2. Methods

#### 2.2.1. Collection and Identification of Plant Samples

Unripen mature fruits of *Annona muricata* were bought from local fruit markets in Lusada and Agbara, Ogun state, Nigeria. Fresh samples of the healthy fruits and leaves were deposited in the Forestry herbarium of the Forestry Research Institute of Nigeria (FRIN), Ibadan, Oyo State, Nigeria. The plant samples were identified and authenticated with voucher referencing Number FHI. 110177.

#### 2.2.2. Preparation of Extracts

The fruits were washed in clean water and peeled manually with knife to remove the epicarp (fruit-skin) which was blended and extracted with 95% ethanol in order to arrive at desired polar compounds in line with the project design. Four other solvents were employed on both the leaves and fruit-skin of this plant beyond the scope of this article. The extract was sieved first with eight-layered muslin cloth and then with a vacuum membrane filter and concentrated in a rotary evaporator at 55–60°C under reduced pressure [14].

#### 2.2.3. Qualitative Phytochemical Screening

Twelve selected phytochemical screening tests were carried out according to methods previously described on the crude extracts for the detection of the following compounds: alkaloids, anthocyanins & betacyanins, coumarins, flavonoids, glycosides, phenols, quinones, saponins, steroids, tannins, terpenoids, and triterpenoids. Prior to the screening, the extracts were dissolved in their respective solvent [15–19].

#### 2.2.4. Quantitative Phytochemical Screening

*Extract Fractionation and Partial Purification.* A designated weight (60.20 g) of ESA was re-dissolved in ethanol and then partitioned by batch into hexane, ethyl acetate and aqueous fractions. Due to the limited amount of crude extract available, 246. 90 g of the bioactive ethanolic fruit-skin extract of the plant was directly subjected to bioactivity-guided purification in the column chromatography. The first column (120 cm in length by 10 cm in diameter) employed in the separation of the crude extract was carefully loaded with dry silica gel in proportion to the extract (10 : 1; ESA: SG) to be separated, followed by the dried extract. Another layer of silica gel was added followed by a layer of cotton wool to prevent the gel and consequently the extract from being displaced. After ensuring that there is no airspace in the loaded column by tapping the sides of the column for the content to settle and stack in properly, the first solvent, 100% chloroform was added to the column to wash the extract thereby eluting nonpolar compounds. Subsequently, the column was eluted with 80 : 1, 50 : 1, 20 : 1, 5 : 1, 1 : 2, 100% methanol and water [20]. Sub-fractions were analysed on thin layer chromatography plates as well as in HPLC [21, 22].

#### 2.2.5. Antioxidant Studies

*Diphenyl-1-Picrylhydrazyl (DPPH) Radical Scavenging Activity Determination.*The scavenging activity of *A. muricata* extracts against diphenyl-1-picrihydrazyl was determined by working with appropriate dilutions of varying concentrations of the extracts (0, 120, 240, 360, 480, 600, and 840 *µ*g/mL) of the sample were mixed with 2 ml of DPPH (0.1 mM) solution and incubated at room temperature in the dark for 60 minutes. Thereafter the absorbance of the sample (AS) and control (AC) was read at 517 nm. Diphenyl-1-picryhydrazyl radical scavenging activity (%) = [(*AC* − *AS*)/(*AC*)] × 100 [23].

#### 2.2.6. Determination of Lipid Peroxidation

Cytosolic and mitochondrial lipid peroxide formation was assayed for by adding mitochondrial and cytosolic samples to test tubes along with varying volumes of the plant extract and finally making it up to 1 mL with distilled water. Ferrous sulphate (0.05 mL of 0.07 M) was added and incubated at room temperature for 30 min; 1.5 mL of 20% acetic acid (pH 3.5) and 1.5 mL of TBARS reagent was added and the test tubes were incubated at 95°C for 60 minutes. After cooling, 5 mL of butan-1-ol was added to each tube and centrifuged at 3000 rpm for 10 minutes and the absorbance of the upper layer was measured at 632 nm [24–27]. Inhibition of lipid peroxidation by the extract is calculated thus:

(1) $$\begin{array}{c} \frac{\left(\text{Absorbance of control}-\text{Absorbance in presence of extract}\right)}{\text{Absorbance of control}}\times 100\%. \end{array}$$

#### 2.2.7. Isolation of Rat Liver Mitochondria

The animals were sacrificed by cervical dislocation and the livers were excised, weighed, washed with homogenizing buffer (210 mM mannitol, 70 mM sucrose, 5 mM HEPES-KOH, pH 7.4 and 1 mM EGTA), minced, homogenized on ice in a Porter-Elvehjem glass homogenizer and centrifuged to isolate the low ionic strength mitochondria according to the method reported by Adegbite et al. [28]. Mitochondrial protein was determined with bovine serum albumin as standard [29].

#### 2.2.8. Determination of Mitochondrial Membrane Permeability Transition

Mitochondrial membrane permeability transition as well as mitochondria swelling were determined by measuring the changes in absorbance of mitochondrial suspension at 540 nm in the presence and absence of triggering agent (calcium chloride) in a spectrum lab 752 s UV/visible spectrophotometer according to the method of Lapidus and Sokolove [30]. Swelling was measured as decrease in absorbance within the time space of 12 minutes. The temperature was maintained at 37°C and the swelling rate quantified at A540/min/mg [28].

#### 2.2.9. Assay for Cytochrome C Release

The reaction mixtures from the mitochondrial membrane permeability assays were stored in eppendorf tubes, centrifuged and the supernatant containing the extramitochondrial cytochrome c was assayed using the rat cytochrome-C (Cyt-C) ELISA kit. Dilutions of the standard solution were made with concentrations 24 ng/mL, 12 ng/mL, 6 ng/mL, 3 ng/mL, and 1.5 ng/mL respectively. The absorbance was measured at 450 nm within 10 minutes of adding the stop solution [31].

## 3. Discussion

**Figure 2:**
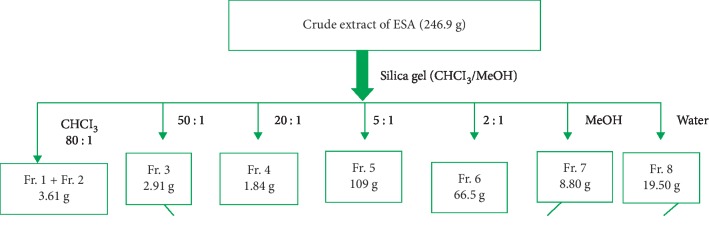
Chart showing the chromatographic separation of the crude 95% ethanol fruit-skin extract of *Annona muricata.*

**Figure 3:**
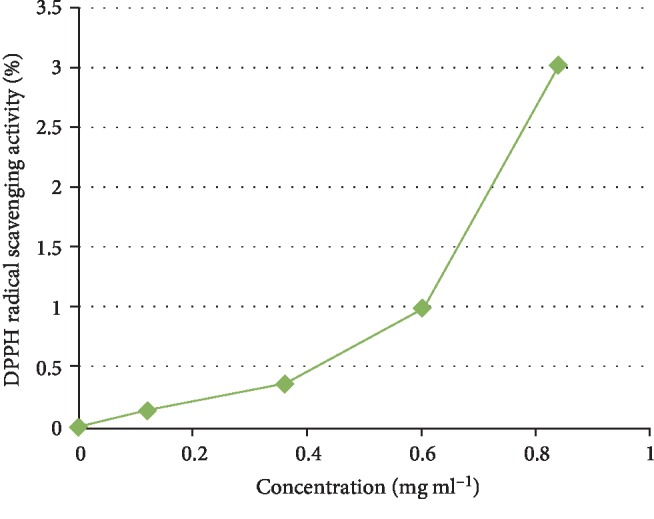
Diphenyl-1-picryhydrazyl radical scavenging activity of ethanol fruit-skin extract of *Annona muricata.*

**Figure 4:**
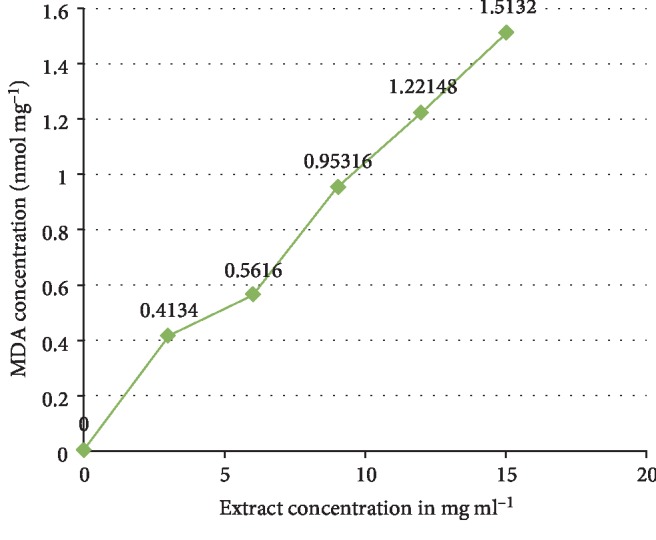
Extent of lipid peroxidation in the hepatocytosolic fraction *in vitro.*

**Figure 5:**
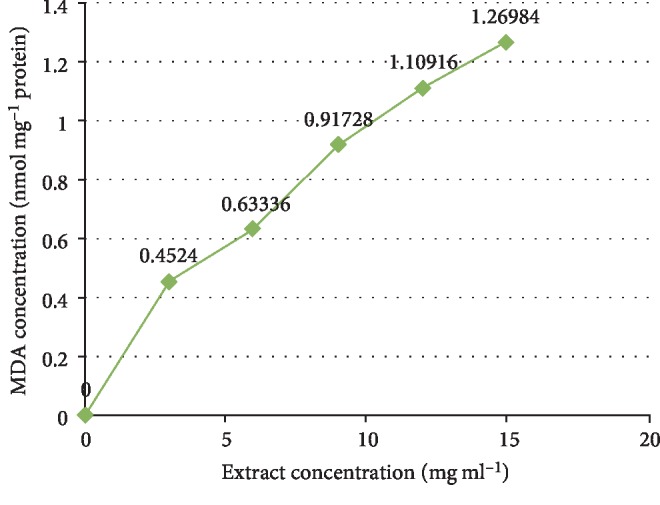
Extent of lipid peroxidation in hepatic mitochondrial fraction *in vitro.*

**Figure 6:**
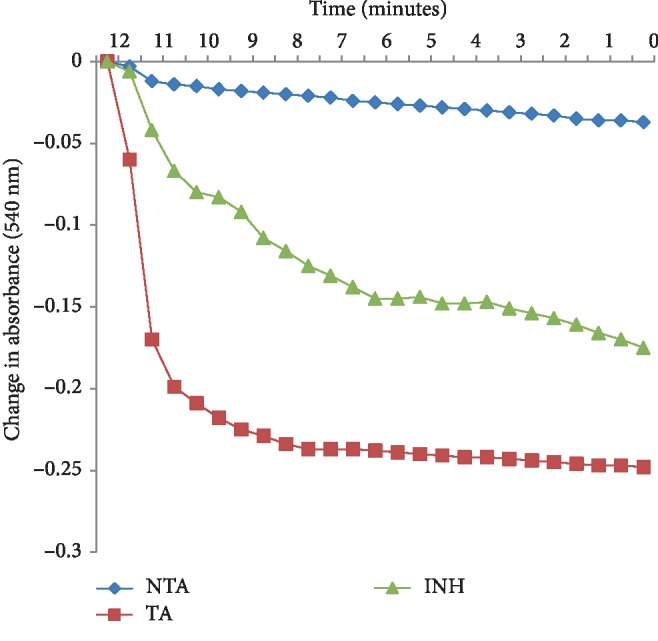
Changes in absorbance (540 nm) over 12 min of assessing mitochondrial permeability transition in rat liver mitochondria (control). NTA: no triggering agent; TA: triggering agent; INH: inhibitor (spermine).

**Figure 7:**
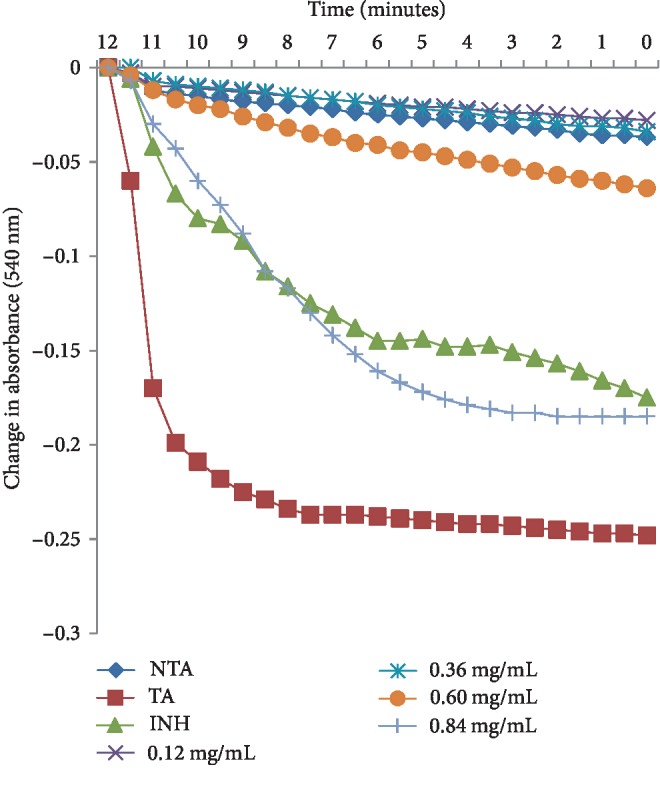
Changes in absorbance (540 nm) for 12 minutes of assessing mitochondrial permeability transition in rat liver mitochondrial fraction treated with varying concentrations of ethanol fruit-skin extract of *Annona muricata* in the absence of calcium. NTA: no triggering agent; TA: triggering agent; INH: inhibitor (spermine).

**Figure 8:**
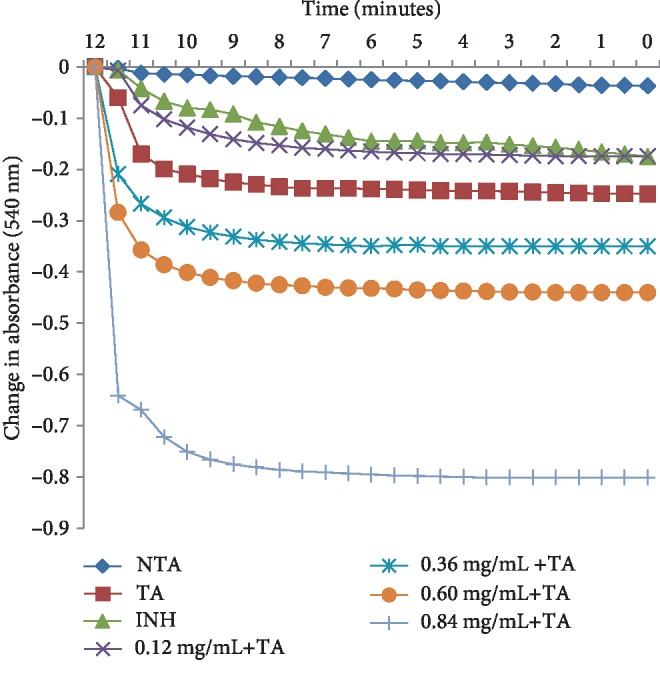
Changes in absorbance (540 nm) for 12 minutes of assessing mitochondrial permeability transition in rat liver mitochondria treated with varying concentrations of ethanol fruit-skin extract of *Annona muricata* in the presence of calcium. NTA: no triggering agent; TA: triggering agent; INH: inhibitor (spermine).

**Figure 9:**
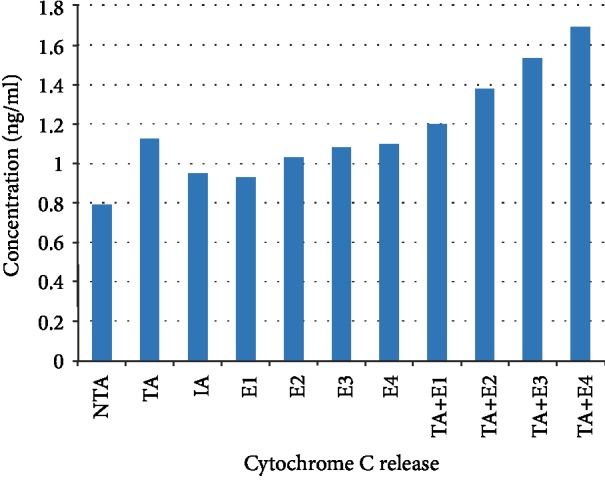
Release of cytochrome c.

The phytochemical study of ESA (Table 1) revealed the presence of alkaloids, antocyanins and betacyanins, coumarins, flavonoids, phenols, quinones, saponins, steroids, tannins, and terpenoids which is in consonance with the findings of Gavamukulya et al. [32]. The constituent groups of phytochemical in these extracts are highly medicinal and have been reported to be active against several disease conditions ranging from bacterial infections to cancer as earlier reported by Solomon-Wisdom et al., [33–35]. Terpenoids constitute the largest group of natural products and are responsible for the characteristic odour of different parts of *A. muricata* plant. Partial purification of ESA gave insight and direction to further studies (Figure 2). Most recently, Kuhnert et al. [36] isolated ten terpenoids from a named fungus, five of which showed moderate cytotoxic activity. In the same vein, Li et al. [37] has reported the potent inhibitory and the apoptotic-stimulating effects of terpenoids on PC-3 prostate cancer cells. The extract exhibited very low (0.14%, 0.36%, 0.99%, and 3.01%) diphenyl-1-picryhydrazyl (DPPH) radical scavenging activity at 0.12, 0.36, 0.60, and 0.84 mg mL^−1^ concentrations respectively as shown in Figure 3, compared to ascorbic acid with 96.87 % activity as reported by Adebayo et al. [38]. Also to further buttress the reduced availability of antioxidants in the crude extract of ESA, the extract at 3.0, 6.0, 9.0, 12.0, and 15.0 mg mL^−1^ respectively induced lipid peroxidation in a concentration-dependent manner on both the cytosolic (0.4134, 0.5616, 0.9532, 1.2215, and 1.5132 nmol mg^−1^ protein) and mitochondrial (0.4524, 0.6334, 0.9173, 1.1092, and 1.2698 nmol mg^−1^ protein) hepatocyte fractions *in vitro* (Figures 4 and 5). Mitochondrial lipid peroxidation has been implicated in oxidative stress triggered apoptosis and mitochondria are the major source of reactive oxygen species (ROS) owing to cellular respiration. The low DPPH radical scavenging activity demonstrated by fruit-skin ethanol extract of *Annona muricata* is possibly as a result of low concentration of available antioxidants such as flavonoids and polyphenols, which is evident in the high IC50 value of 1570 *μ*g mL^−1^compared to ascorbic acid (~90%) which can be as low as 9.3 *μ*g mL^−1^. This result is very closely related to the findings of Lee et al. [39] on the fruit skin ethanol extracts of *Annona muricata* under varying extraction conditions with IC50 of 1179.96 *μ*g mL^−1^. Kuhnert et al. [36] also assessed ethyl acetate and n-butanol fractions of *Annona muricata* leaf extract and reported very low IC50 values 4.3 ± 0.7 and 9.3 ± 0.8 *μ*g mL^−1^ respectively; this finding further validates the fact that leaves and seeds of *Annona muricata* possess higher antioxidant potential than the fruit-skin; although extraction solvents also significantly influence phytochemicals present in a given extract [40]. Gaikwad et al. [41] noted that IC_50_ values have been calculated for thousands of plant extracts and the findings of this research becomes very pertinent as it adds to the growing list [41]. The higher antioxidant activities of the leaves and seeds of *Annona muricata* has been attributed to the presence of both enzymic and non-enzymic antioxidants, including catalase and superoxide dismutase as well as tocopherol and ascorbic acid as earlier brought to the fore by Baskar et al. [42]. When ROS are generated in excessive concentrations due to a pathological condition, mitochondrial-mediated apoptosis can be triggered and this is hallmarked by the opening of the mitochondrial permeability transition (MMPT) pore [28]. Excessive calcium ion concentration in the mitochondria due to calcium overload in the cell, have also been implicated in mitochondrial swelling. This study revealed that ESA at tested concentrations with the exception of the highest concentration (0.84 mg mL^−1^) did not induce MMPT pore opening in the absence of CaCl_2_, (triggering agent) as shown in Figures 6 and 7. However, 0.84 mg mL^−1^ ESA induced MMPT pore opening by 129% (Table 2). Furthermore, the extracts showed no inhibitory activity in the presence of the triggering agent as it induced pore opening beyond the extent of the triggering agent with induction fold of 116.25, 337.50, 450.00, and 897.50 at the respective tested concentrations (Table 2 and Figure 8). The anti-apoptotic activity demonstrated by the lower three of the four concentrations of the fruit-skin ethanol extract of *Annona muricata* tested connotes the absence or reduced concentration of cytotoxic compounds within that concentration range and below. This becomes valid as the highest concentration tested in this study, 0.84 mg mL^−1^ induced the opening of the mitochondrial membrane permeability transition pore leading to cytochrome c release and cell death. Moghadamtousi et al. [11] carried out a thorough study on the ethyl acetate leaf fractions of *Annona muricata* and concluded that the fraction was cytotoxic to HT-29 and HCT-116 colon cancer cells with attendant suppressive effect on cell line proliferation. Furthermore, the cell death mechanism was reported to have occurred via the mitochondrial-mediated pathway. This latter magnanimous findings validates the outcomes of the current study, highlighting the fact that the fruit-skin ethanol extract of *Annona muricata* at concentrations above 840 *μ*g mL^−1^ can induce apoptosis; and this possibly via the mitochondrial pathway, since cytochrome c release also increased in a concentration-dependent manner. The cell death pathway for the fruit-skin ethanol extract could possibly have been characterized by the up-regulation of proapoptotic Bax protein and down-regulation of anti-apoptotic bcl-2 protein, which then drops the mitochondrial membrane potential leading to membrane disruption and subsequent release of cytochrome c; ultimately causing the activation of caspase-9 (of the intrinsic/mitochondrial pathway of cell death) as well as caspases 3, 6, and 7 which are the executioner caspases that eventually facilitates the downstream morphological changes involved in apoptotic cell death. It is also noteworthy that ESA at all concentrations tested, did not reverse the opening of the MMPT pore in rat liver mitochondria in vitro in the presence of calcium like spermine; whereas the lower three concentrations did exhibit inhibition of the pore in the absence of calcium. At lower concentration below 800 *μ*g mL^−1^, ESA could help ameliorate oxidative stress within the cell by protecting the integrity of the cell membrane as equally reported by Vijayameena et al. [43]. This suggests that ESA at lower concentrations possibly contains certain phytochemicals such as tocopherol and ascorbic acid in minute concentrations which effect could be overshadowed by other phytochemicals such as phytol and phytol acetate which has been purified from *Annona muricata* leaves at elevated concentrations. These latter phytochemicals have been reported to induce apoptosis in cancer cell lines [44]. Furthermore, it would be very interesting to note that ethyl acetate leaf fractions of *Annona muricata* suppressed colon cancer growth by arresting the G1 phase of the cell cycle and blocked migration and invasion of cancer cell lines; this remarkable feature of *Annona muricata* ought to be given priority study and intense comprehensive research in order to develop viable therapeutic remedies to life-threatening conditions staring the face of mankind worldwide [44]. Padma et al. [45] demonstrated that extracts from the stem bark of *A. muricata* reduced lipid peroxidation in the brain and liver of rats subjected to cold-immobilized stress. Several other researchers have also reported how several plant parts of *Annona muricata* such as leaves, pulp, stem bark, and root bark have significantly reduced lipid peroxidation *in vitro* and *in vivo* [39, 45–47]. All these conclusions do not agree with the findings of this study as lipid peroxidation graphs both for extract incubation with the cytosolic and mitochondrial fraction of rat hepatocytes presented positive slopes; meaning lipid peroxidation was induced in a concentration-dependent manner at concentrations tested. This can as well be understood from the proapoptotic feature also demonstrated by the highest concentration tested extract in opening the MMPT pore, which may be as a result of oxidative stress as observed by Moghadamtousi et al. [11]. Also noteworthy is the fact that the induction fold of the MMPT pore opening corresponded with the concentrations of cytochrome c released (Figure 9) both in the absence and in the presence of the triggering agent. There is an active principle previously purified from *Annona muricata* which triggers apoptosis via the mitochondrial pathway; this annonaceous acetogenin called bullatacin is a potent antitumour compound that mediates cytochrome c release by reducing both intracellular cyclic AMP and cyclic GMP as well as inhibiting NADH: Ubiquinone oxidoreductase (complex I) of the electron transfer system. Bullatacin has been identified to be highly cytotoxic against multidrug-resistant human mammary adenocarcinoma cells; which explains the concentration-dependent cytochrome c release observed in the extract tested in this study [48–52].Nonetheless, Correa-Gordillo et al. [53] came up with a review of the antioxidant capacity of *Annona muricata* and categorically stated that soursop did not contain high activity or concentration of antioxidants in leaves, fresh/frozen pulp, juice as well as wine from the plant. This review perfectly supports our finding in this study as regards the antioxidant feature of fruit-skin ethanol extract of *Annona muricata* [54, 55].

## 4. Conclusion

Suffice to say that there are extremely few reports on the fruit-skin (epicarp) of *A. muricata*; this study proposes that the fruit-skin ethanol extract of *Annona muricata* at concentrations above 840 *μ*gmL^−1^ do not possess adequate antioxidants that could function to inhibit mitochondrial-mediated apoptosis, rather they possibly contain potent bioactive phytochemicals that induces apoptosis. Further studies are ongoing on other solvent-extracts of the fruit-skin and leaves of *A. muricata* in a bid to purify and identify potent bioactive compounds against certain bacterial agents of specified acute respiratory infections (ARIs). Additional studies should be carried out on different solvent extractions of the fruit skin of *A. muricata* to elucidate the specific mechanism of cytotoxicity, as this has the potential of opening up new areas in the discovery of nature-friendly therapeutics for pathological conditions like cancer.

## Figures and Tables

**Table 1:** Phytochemical study of ESA.

Test	Status
Tannins	+
Flavonoids	+
Terpenoids	+
Phenols	+
Steroids	+
Amino acids	−
Alkaloids	+
Glycosides	−
Quinones	+
Saponins	+

(+) Denotes presence of phytochemical; (−) Denotes absence or no trace of phytochemical in the extract tested; ESA: ethanol skin extract of *Annona muricata.*

**Table 2:** Induction fold for MMPT pore opening.

Sample	Induction fold (%)
NTA	0.0000
TA	467.50
INH	118.75
E1 (0.12 mg/mL)	0.0000
E2 (0.36 mg/mL)	0.0000
E3 (0.60 mg/mL)	0.0000
E4 (0.84 mg/mL)	128.75
TA + E1	116.25
TA + E2	337.50
TA + E3	450.00
TA + E4	897.50

ESA: ethanolic fruit skin of *Annona muricata,* NTA: no triggering agent, TA: triggering agent, INH: inhibitor, E1–E4: extract at respective concentration.

## Data Availability

The data used to support the findings of this study are included within the article.
